# The SDF-1/CXCR4 axis regulates migration of transplanted bone marrow mesenchymal stem cells towards the pancreas in rats with acute pancreatitis

**DOI:** 10.3892/mmr.2014.2053

**Published:** 2014-03-14

**Authors:** JIAN GONG, HONG-BO MENG, JIE HUA, ZHEN-SHUN SONG, ZHI-GANG HE, BO ZHOU, MING-PING QIAN

**Affiliations:** Department of General Surgery, Shanghai Tenth People’s Hospital, Tongji University of Medicine, Shanghai 200072, P.R. China

**Keywords:** mesenchymal stem cells, migration, stromal cell-derived factor-1, CXC chemokine receptor-4, acute pancreatitis

## Abstract

Stromal cell-derived factor-1 (SDF-1) and its receptor, CXC chemokine receptor-4 (CXCR4), are important regulators in the migration of bone marrow mesenchymal stem cells (BMSCs). However, the mechanisms underlying this effect in acute pancreatitis (AP) have not been investigated. In this study, BMSCs were identified by specific cell surface markers and differentiation potentials, and labeled with chloromethylbenzamido-1,1′-dioctadecyl-3,3,3′,3′-tetramethylindocarbocyanine perchlorate (CM-Dil) for *in vivo* cell tracking. AP was induced by retrograde infusion of sodium taurocholate into the common bile duct in rats. The expression of SDF-1 in the injured pancreas was determined by immunohistochemistry and western blot analysis. BMSCs were incubated with or without anti-CXCR4 antibody and the contribution of SDF-1 to the migration of BMSCs was investigated. Our results demonstrated that the expression of SDF-1 was significantly increased in the injured pancreas, and that these levels peaked on days 5–7 and began to decrease on day 10. SDF-1 induced a dose-dependent migration of BMSCs in an *in vitro* transwell migration assay, which was almost completely blocked by AMD3100 (CXCR4-specific antagonist) or anti-CXCR4 antibody. In addition, by encouraging the migration of CM-Dil-labeled BMSCs, the SDF-1/CXCR4 axis facilitated the repair of the injured pancreas. This effect was inhibited by the anti-CXCR4 antibody. Taken together, these results indicate that the interaction of locally produced SDF-1 with CXCR4 on BMSCs, has an important regulatory role in the migration of BMSCs towards the injured pancreas in AP.

## Introduction

Acute pancreatitis (AP) is a common acute abdominal disease, and ~10–20% of AP patients develop severe acute pancreatitis (SAP)(1). SAP is associated with poor prognosis and mortality rates as high as 15–20% (2). Current conservative therapies including inhibition of pancreatic juice synthesis and secretion, prophylactic use of antibiotics and nutritional support, are most frequently utilized in the treatment of SAP in clinical practice, however these strategies are of poor efficacy.

Mesenchymal stem cells (MSCs) are a type of adult stem cell in humans, characterized by their low immunogenicity and multiple differentiation potentials. Under certain conditions, MSCs are able to differentiate into various tissue cell types, including myocardial cells, endothelial cells and insulin-secreting cells (3–5). Several studies have demonstrated that MSCs are involved in the pathogenesis of AP, revealing in their investigations that transplanted MSCs have the capacity to relieve the severity and improve the prognosis of SAP (6–8). While it is understood that MSCs have the ability to migrate into the damaged organ, the mechanism of this effect is largely unknown.

Stromal cell-derived factor-1 (SDF-1) is a member of the CXC chemokine family, and binds to its highly specific corresponding receptor, CXC-chemokine receptor 4 (CXCR4), forming a complex from which it regulates numerous physiological functions. Previous studies have reported that the SDF-1/CXCR4 axis not only is involved in the invasion and metastasis of malignant tumors (9,10), however is also critical in the migration of stem cells. Du *et al* (11) identified that SDF-1 is able to significantly enhance the migration and differentiation of periodontal ligament stem cells to repair damaged periodontal tissues. Similarly, Theiss *et al* (12) demonstrated that the SDF-1/CXCR4 axis, which promoted stem cell homing, may be important in the treatment of myocardial infarction. Furthermore, SDF-1 expression in tissues appears to be significantly increased under hypoxic conditions, including during myocardial ischemia (13), cerebral ischemia (14) and renal failure (15). Thus, we hypothesize that SDF-1/CXCR4 axis may also promote the migration of MSCs towards the injured pancreas in AP.

In this study, SDF-1 expression in the pancreas of AP rats, and the *in vitro* effect of SDF-1 on the migration of bone marrow mesenchymal stem cells (BMSCs), was investigated. In addition, the BMSCs were transplanted into an *in vivo* rat model of AP via the tail vein to examine the role of SDF-1/CXCR4 axis in the migration of BMSCs in the injured pancreas.

## Materials and methods

### Induction of AP

All animal procedures were conducted according to the Shanghai Laboratory Animal Ordinance and approved by the Ethics Committee of Shanghai Tenth People’s Hospital (Tongji University, Shanghai, China). Sprague-Dawley (SD) rats were purchased from Shanghai SLAC Laboratory Animal Co., Ltd. (Shanghai, China). These animals were housed under standardized conditions in a 12 h dark/light cycle with free access to water and food. Rats were fasted overnight preoperatively. Following being anesthetized by intraperitoneal injection of 3% pentobarbital, a 2 cm midline laparotomy was performed and the first loop of the duodenum and the pancreas were separated. A blunt fine catheter was then introduced into the bile-pancreatic duct and 3% sodium taurocholate (1 ml/kg; Sigma-Aldrich, St. Louis, MO, USA) was retrogradely injected into the common bile duct within 60 sec. Leakage of sodium taurocholate was prevented by temporary ligation of the distal bile duct with a 9-0 prolene suture, while the proximal bile duct was temporarily occluded with a microvessel clip. Following infusion, the blunt fine catheter, the microvessel clip and the suture were removed to allow the physiological flow of bile. The puncture site on the duodenum was closed with a 6-0 prolene suture and the wound with a 4-0 prolene suture.

### Isolation, culture and identification of rat BMSCs

Four week-old SD rats were sacrificed and the primary BMSCs were isolated from the femurs and tibias under a sterile condition. Following flushing the bone marrow cavity with DMEM-LG medium (Invitrogen Life Technologies, Grand Island, NY, USA) and removing large tissues with a 200-mesh nylon filter, the isolated bone marrow cells were cultured in DMEM-LG medium supplemented with 10% fetal bovine serum (FBS; Invitrogen Life Technologies), 1% penicillin and streptomycin (Invitrogen Life Technologies) at 37°C in an environment with 5% CO_2_. The medium was refreshed every 3 days and cells were harvested by multiple digestions and passaged when cell confluence reached >80%. Cells of the fifth passage were collected and subjected to flow cytometry analysis. These cells were maintained in adipogenic, osteogenic and chondrogenic differentiation medium (Cyagen Biosciences, Sunnyvale, CA, USA) followed by Oil Red O staining, Alizarin Red S staining and Toluidine Blue staining, respectively. The surface markers were detected using a BD FACSC™ II flow cytometry system (BD Biosciences, San Jose, CA, USA). The antibodies utilized in this analysis included anti-CD44-PE, anti-CD73-APC, anti-CD90-FITC, anti-CD105-PerCP-Cy5.5, anti-CD11b-PE, anti-CD19-PE, anti-CD34-PE and anti-CD45-PE (BD Biosciences). Isotype-matched antibodies were used as controls. Cells were harvested between 3–5 passages.

### Immunohistochemistry and western blot analysis

Following AP induction, pancreatic tissues were obtained on days 1, 3, 5, 7 and 10, and normal pancreatic tissues were collected as controls. Immunocytochemistry and western blot analysis were employed to detect the SDF-1 expression. Five SD rats were used at each time point.

After preparation of the paraffin sections, Histostain-Plus kit (DAB, Rabbit; Invitrogen Life Technologies) was used to perform immunohistochemistry. Following conventional dehydration, antigen retrieval and antigen blocking, sections were incubated with rabbit anti-SDF-1 antibody (1:50; Santa Cruz Biotechnology, Inc., Santa Cruz, CA, USA) overnight at 4°C in a wet box. Then, the biotinylated secondary antibody and streptavidin conjugated horseradish peroxidase (HRP) were independently incubated with these sections for 10 min at 37°C. Following staining with DAB, the sections were counterstained with hematoxylin and then dehydrated and mounted with neutral resin.

Pancreatic tissues were added to liquid nitrogen and crushed. The total protein was extracted with the conventional method and was followed by determination of protein concentration with bicinchoninic acid method. Subsequently, 30 mg of proteins were subjected to 12% polyacrylamide-SDS gel electrophoresis and electroblotted onto nitrocellulose membranes, which were then incubated with rabbit anti-SDF-1 antibody (dilution, 1:200; Santa Cruz Biotechnology, Inc.) overnight at 4°C following blocking with 5% skimmed milk for 1 h. Incubation with the secondary antibody at room temperature for 2 h was conducted and then visualization was performed, and the protein bands were scanned (Gene Company Limited, Hong Kong, China) and analyzed using Odyssey 3.0 analysis software (Li-Cor Bioscience, Lincoln, NE, USA).

### Immunofluorescence staining

The CXCR4 expression profile on BMSCs was assessed by immunofluorescence staining. Cells were collected and fixed in 4% paraformaldehyde for 20 min. Following treatment with 0.5% Triton X-100 and 1% bovine serum albumin (BSA), cells were incubated with the rabbit anti-CXCR4 antibody (dilution, 1:50; Santa Cruz Biotechnology, Inc.) overnight at 4°C. After washing with PBS, cells were incubated with the fluorescein isothiocyanate (FITC)-conjugated secondary antibody for 30 min at 37°C followed by DAPI (Sigma-Aldrich) staining for 2 min, for nuclear counterstaining. The sections were mounted and then observed under a Zeiss LSM 710 confocal microscope (Carl Zeiss, Oberkochen, Germany).

### Transwell migration assay

The migration assay was designed using transwell plates (Corning Costar, Cambridge, MA, USA) that were 6.5 mm in diameter with 8 μm pore filters. In the chemotaxis assay, the upper chambers were loaded with 5×10^4^ BMSCs in 200 μl of DMEM containing 0.1% BSA, and the lower chambers with 500 μl of DMEM containing 10% FBS and SDF-1 at different concentrations (Peprotech, Inc., Rocky Hill, NJ, USA). SDF-1 at 0, 10, 50, and 100 ng/ml was used according to the manufacturer’s instructions. In the chemotaxis inhibition assay, the upper chambers were inoculated with 5×10^4^ BMSCs that were incubated with rabbit anti-CXCR4 antibody (10 μg/ml; Santa Cruz Biotechnology, Inc.) or AMD3100 (10 μg/ml; Sigma-Aldrich), an antagonist of CXCR4, for 2 h at 37°C. The lower chambers were added with 500 μl of DMEM containing 10% FBS and SDF-1 at optimal concentration. Following incubation for 15 h, cells in the upper chamber were removed and the membranes were fixed in 4% paraformaldehyde for 20 min. The cells that migrated to the lower side of the filter were stained with 0.1% crystal violet for 10 min and then observed under a light microscope. Crystal violet was dissolved in 300 μl of 33% acetic acid and the absorbance at 573 nm was measured with an enzyme-labeling measuring instrument (Gene Company Limited).

### Animal experiments

To trace BMSCs *in vivo*, cells were labeled with chloromethylbenzamido-1,1′-dioctadecyl-3,3,3′,3′-tetramethylindocarbocyanine perchlorate (CM-Dil; 1 μg/ml; Invitrogen Life Technologies) at 37°C for 30 min. Several studies have demonstrated that labeling cells with CM-Dil does not affect cell viability, proliferation or differentiation. The labeling efficiency was determined by fluorescence microscopy (16,17).

Rats were randomly divided into four groups: the control group (n=15), the AP group (n=15), the BMSCs group (n=15) and the anti-CXCR4 group (n=15). Rats were sacrificed at days 1, 4 and 7 (n = 5/time point). Rats in the anti-CXCR4 group received an injection of CM-Dil-labeled BMSCs (1×10^7^ cells/ml/kg) and were pretreated with anti-CXCR4 antibody via the tail vein. Rats in the BMSC group received an injection of CM-Dil-labeled BMSCs (1×10^7^ cells/ml/kg). In the control and AP group, rats were treated with normal saline of equal volume. Following sacrificing, the pancreas and blood were collected. Each pancreatic tissue was divided into two sections: one was fixed in 4% paraformaldehyde for immunohistochemistry and HE staining, and the other was prepared for the frozen sections, in order to detect the migration of BMSCs towards the pancreatic tissues by confocal microscopy (Zeiss). The images were captured at five randomly selected fields at a high magnification and the migrated CM-Dil-labeled BMSCs (red cells) were calculated. Blood was centrifuged and supernatant collected for serum amylase analysis.

### Histology and serum amylase

Paraffin sections were prepared for histopathological examination with conventional HE staining. In order to quantify the pancreatic injury, 20 microscopic fields were randomly selected as previously described (18). In brief, the edema, inflammatory cell infiltration and acinar necrosis were divided into four grades (edema: 0 = absent, 1 = focally lobular edema, 2 = diffusely lobular edema and 3 = acini edema and separated; inflammatory cell infiltration: 0 = absent, 1 = infiltration around ductal margins, 2 = infiltration into <50% of lobules and 3 = infiltration of >50% of lobules; acinar necrosis: 0 = absent, 1 = necrosis in <5% of lobules, 2 = necrosis in 5–20% of lobules and 3 = necrosis in 20–50% of lobules).

The amylase activity assay kit (Biovision, Mountain View, CA, USA) was used to determinate the serum amylase activity. The nitrophenol standard curve was delineated. Then, the optical density (OD) immediately prior to reaction (ODT_0_) and at 10 min following reaction (ODT_1_) was measured at 405 nm. The difference of OD was calculated as follows: ΔOD = ODT_1_ − ODT_0_, and the concentration of nitrophenol was obtained according to the standard curve and then converted into the amylase activity.

### Statistical analysis

Statistical analysis was performed using SPSS version 14.0 for Windows (SPSS, Inc., Chicago, IL, USA). Data were presented as the mean ± standard deviation (SD) and were analyzed with a Student’s t-test. A value of P<0.05 was considered to indicate a statistically significant result.

## Results

### Characterization of BMSCs

**Figure 1:**
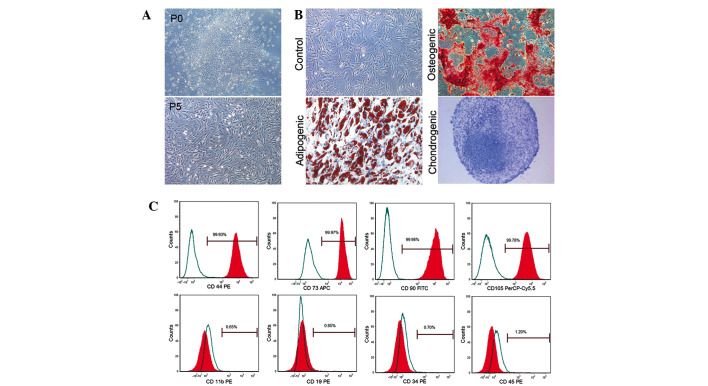
Characterization of rat BMSCs. (A) Spindle-shaped cells formed a colony in primary cells and cells after passaging several times (P5) remained with their fibroblast-like morphology (magnification, ×40); (B) Differentiation potentials of BMSCs: osteogenic differentiation (Oil Red O staining; magnification, ×100), adipogenic differentiation (Alizarin Red S staining; magnification, ×200) and chondrogenic differentiation (Toluidine Blue staining; magnification, ×100). (C) Several markers of BMSCs identified by flow cytometry. BMSCs, bone marrow mesenchymal stem cells.

A large number of nucleated cells were attached to the bottom of the plates under a light microscope. These cells demonstrated fibroblast-like morphology on days 5–6 and cell colonies were found on days 7–10 (Fig. 1A). These cells gradually aged after passaging 6–7 times, as identified by an increased cell size, intracellular fine particulate matter and a decrease in cell proliferation. These cells demonstrated excellent multi-lineage plasticity and successfully differentiated into adipocytes, osteoblasts and chondrocytes following *in vitro* adipogenic, osteogenic and chondrogenic differentiation induction for 21, 14 and 28 days, respectively (Fig. 1B). These cells were identified by BMSC markers. Flow cytometry identified that these cells were markedly positive for CD44, CD73, CD90 and CD105 (99.93, 99.97, 99.98, 99.78%, respectively) but negative for CD11b, CD19, CD34, and CD45 (0.65, 0.85, 0.70 and 1.20%, respectively; Fig. 1C).

### SDF-1 expression in damaged pancreas

**Figure 2:**
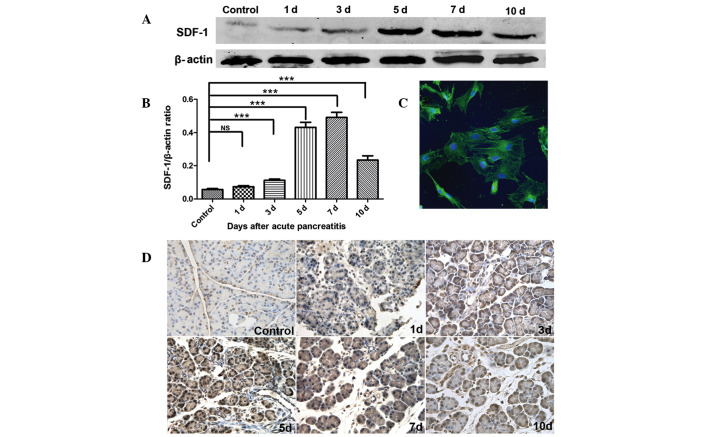
SDF-1 expression in pancreatic tissues and CXCR4 expression in BMSCs. (A) SDF-1 protein expression in damaged pancreas detected by western blot analysis at different time points. (B) Determination of band density, SDF-1/β-actin ratios were significantly higher on days 3, 5, 7 and 10 as compared with the control group (P<0.001); (C) CXCR4 expression on BMSCs was observed by confocal microscopy. Green, CXCR4; blue, DAPI positive nuclei, original magnification, ×500. (D) SDF-1 expression in damaged pancreas detected by immunohistochemistry at different time points. ^***^P<0.001. ns, not significant; BMSCs, bone marrow mesenchymal stem cells; CXCR4, CXC-chemokine receptor 4; SDF-1, stromal cell-derived factor-1.

To investigate the effect of the SDF-1/CXCR4 axis on the migration of BMSCs, the SDF-1 expression in damaged pancreas was measured and observed at different time points following AP induction. As summarized in Fig. 2D, in the control group, pancreatic tissues exhibited a low SDF-1 expression on the cell membrane and in the cytoplasm. The SDF-1 expression was gradually increased following AP induction, peaking on days 5–7, however declining from day 10. The SDF-1 protein expression as determined by western blot analysis was markedly increased in the inflammatory area as compared with the control group, which was consistent with findings in the immunohistochemistry assay. The SDF-1/β-actin ratio on day 7 was the highest, being 8.6-fold greater than that in the control group. The SDF-1/β-actin ratio in AP group on days 3, 5, 7, 10 was significantly different from that in control group (P<0.001; Fig. 2A and B).

### CXCR4 expression on BMSCs

BMSCs were treated with anti-CXCR4 antibody for 2 h and then the CXCR4 expression on BMSCs was detected using confocal microscopy. Five fields were randomly selected for analysis, and the results demonstrated that >90% of BMSCs expressed CXCR4, with certain cells having a particularly high expression (Fig. 2C).

### SDF-1 induces BMSC migration in vitro

**Figure 3:**
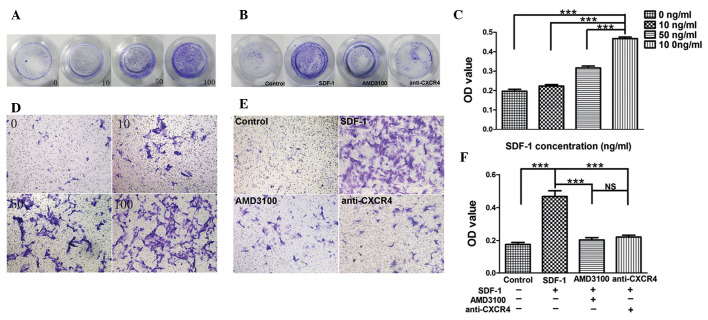
Transwell migration assay. (A) Macroscopic observation of transwell chamber (chemotaxis experiments). (B) Macroscopic observation of transwell chamber (chemotaxis inhibition experiments). (C) Absorption measured at 405 nm (chemotaxis experiments). (D) Migrated BMSCs under a light microscope (chemotaxis experiments). (E) Migrated BMSCs under a light microscope (chemotaxis inhibition experiments). (F) Absorption measured at 405 nm (chemotaxis inhibition experiments). ^***^P<0.001. ns, not significant; SDF-1, stromal cell-derived factor-1; BMSCs, bone marrow mesenchymal stem cells.

Our results revealed that SDF-1 induced BMSC migration in a dose-dependent manner, and that maximum migration was observed following treatment with SDF-1 at 100 ng/ml, which indicates that SDF-1 is important in BMSC migration (Fig. 3A and B). The absorption at 405 nm following treatment with SDF-1 at 100 ng/ml was significantly increased as compared with the other groups (P<0.001; Fig. 3C). In addition, SDF-1-induced migration was markedly inhibited when BMSCs were pretreated with anti-CXCR4 antibody or AMD3100 (Fig. 3D and E). The absorption at 405 nm in the anti-CXCR4 group and AMD3100 group was significantly decreased as compared with the SDF-1 group (P<0.001). However, there was no difference between the anti-CXCR4 group and AMD3100 group (P>0.05; Fig. 3F), which indicate that the inhibitory effect of anti-CXCR4 antibody is similar to that of AMD3100.

### SDF-1/CXCR4 axis promotes BMSCs migration into damaged pancreas and pancreatic repair

**Figure 4:**
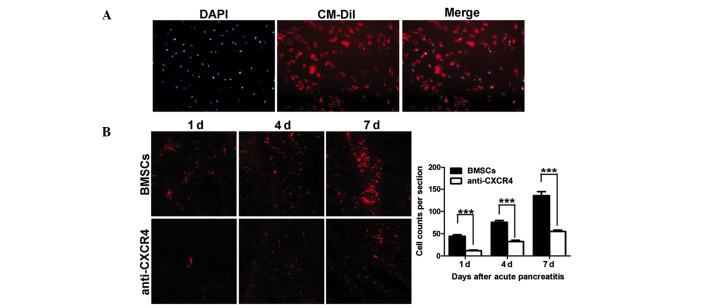
Migration of transplanted BMSCs. (A) CM-Dil-labeled BMSCs. Red, CM-Dil; blue, DAPI positive nuclei. Magnification, ×100. (B) Migration of CM-Dil-labeled BMSCs in the pancreas determined by using confocal microscopy, 25× oil; (C) Cell counting of migrated BMSCs. The number of migrated BMSCs was significantly declined in the anti-CXCR4 group as compared with the BMSCs group at different time points (^***^P<0.001). CM-Dil, chloromethylbenzamido-1, 1′-dioctadecyl-3,3,3′3′-tetramethylindo-carbocyanine perchlorate; BMSCs, bone marrow mesenchymal stem cells; CXCR4, CXC-chemokine receptor 4.

In order to trace the cells *in vivo*, CM-Dil was utilized to label migrating BMSCs. Results demonstrated >95% of BMSCs were successfully labeled by CM-Dil (Fig. 4A). To investigate the effect of CM-Dil-labeled BMSCs in damaged pancreas, frozen sections were prepared as previously described. The results demonstrated CM-Dil positive BMSCs in the BMSCs and anti-CXCR4 groups, however not in the AP and control groups. Concurrently, at each time point, the number of CM-Dil-labeled BMSCs in the anti-CXCR4 group was significantly lowered as compared with the BMSCs group (Fig. 4B). Cells were counted in five randomly selected fields at a high magnification and the total number of cells per section was determined. The results revealed that blocking CXCR4 expression on BMSCs significantly inhibited the migration of BMSCs towards the damaged pancreas (Fig. 4C).

**Figure 5:**
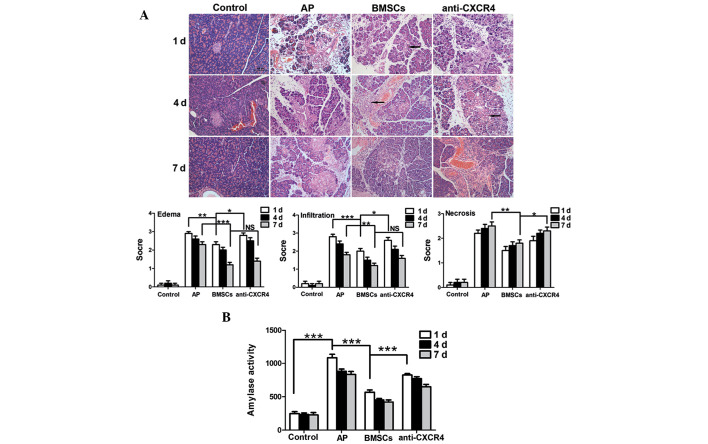
Pancreatic histology and serum amylase activity. (A) Pathology of pancreatic tissues (magnification, ×200), edema and inflammation were significantly improved in the BMSCs group as compared with the control group on days 1 and 7 (P<0.01) and necrosis attenuated on day 7 (P<0.01). Edema and inflammation were improved in the BMSCs group as compared with the anti-CXCR4 group on day 1 (P<0.05) but not on day 7 (P>0.05). Necrosis was more severe in the anti-CXCR4 group than in the BMSCs group on day 7 (P<0.05). A large number of tubular complexes were observed in the BMSCs and anti-CXCR4 groups (as indicated by the arrow). (B) Serum amylase activity: serum amylase activity was significantly higher in the AP group than that in the control group on day 1 (P<0.001), and serum amylase activity was significantly lower in the BMSCs group compared with that in the AP and anti-CXCR4 group (P<0.001). ^*^P<0.05; ^**^P<0.01; and ^***^P<0.001; NS, not significant; d, day; AP, acute pancreatitis; BMSC, bone marrow mesenchymal stem cells; CXCR4, CXC-chemokine receptor 4.

Serum amylase analysis revealed that the amylase activity in the AP group was significantly higher compared with the control group on day 1 (P<0.001). Amylase activity appeared to decrease after BMSCs were transplanted and was significantly decreased as compared with the AP group (P<0.001). Concurrently, serum amylase activity in the anti-CXCR4 group was markedly higher than that in the BMSC group (P<0.001; Fig. 5B). The results of HE staining were consistent with the change in serum amylase activity, and revealed that the BMSCs could reduce pancreatic edema, hemorrhage and necrosis. This effect was compromised following pre-treatment with the anti-CXCR4 antibody. Furthermore, a large number of tubular complexes were observed during pancreatic repair in the BMSC group (Fig. 5A).

## Discussion

BMSCs are specialized adult bone marrow stem cells that have a characteristically potent ability to differentiate. In the present study, BMSCs were obtained from SD rats and cultured. Initially, the cell purity was at a low level. The confounding cells were gradually eliminated by refreshing the medium, controlling the time of trypsin digestion and passaging cells. The cell morphology and results of flow cytometry and differentiation induction were consistent with previously reported data (19,20), according to the characteristics of MSCs (21).

Several studies have demonstrated that SDF-1 is expressed by numerous cells, including vascular endothelial cells (22), acinar cells (23), fibroblasts (24) and cardiomyocytes (13), and that hypoxia upregulates SDF-1 expression. Ceradini *et al* (25) identified that hypoxia upregulated SDF-1 expression by activating hypoxia-inducible factor-1 (HIF-1), to promote the migration of CXCR4-positive progenitor cells into ischemic tissues. Similarly, Lerman *et al* (26) confirmed that hypoxia-induced SDF-1 expression was significantly reduced when the HIF-1 gene was silenced. The pancreatic tissues in AP are ischemic and hypoxic, and SDF-1 expression in the injured pancreas gradually increased over time, peaked on days 5–7 and began to decline on day 10, which confirmed that SDF-1 expression in injured pancreas was significantly higher than those in normal pancreatic tissues. In addition, SDF-1 is secreted by the proliferative acinar cells, vascular endothelial cells and fibroblasts on days 5–7 following AP induction, which attributed to the peak SDF-1 expression. Jung *et al* (7) reported the number of BMSCs migrated to the damaged pancreas in SAP was larger than that in mild AP (MAP). The pancreatic ischemia and hypoxia in SAP were more severe than in MAP, leading to more BMSCs migration with increased SDF-1 expression in SAP rats.

Our *in vitro* investigations demonstrated there was a positive correlation between BMSC migration and SDF-1 concentration. Maximum migration was observed when the SDF-1 concentration was 100 ng/ml, so our results were consistent with data previously reported (13,27). Following neutralization or inhibition of SDF-1 (anti-CXCR4 antibody and AMD3100), the migration of BMSCs was significantly inhibited, which confirms that SDF-1/CXCR4 axis is important in BMSCs migratory behavior.

Numerous studies have reported that BMSCs utilize the SDF-1/CXCR4 axis to migrate to damaged tissues in several pathological conditions, including myocardial ischemia (13), wound area (28), bone fracture (29) and cerebral ischemia (30). The SDF-1/CXCR4 axis links stem cell migration to cell homing (31). This study investigated whether BMSCs migrated to the damaged pancreas in AP and whether the SDF-1/CXCR4 axis was critical in this process. Firstly, our results revealed >90% of BMSCs expressed CXCR4 as demonstrated in immunofluorescence staining, which was consistent with previously reported data (28,32). In our *in vivo* investigations, the BMSCs successfully migrated to the damaged pancreas in the SAP group. Of note, this effect was significantly inhibited when the SDF-1/CXCR4 axis was blocked. Thus, we hypothesize that the SDF-1/CXCR4 axis is a key mediator in BMSC migration to the damaged pancreas. However, CM-Dil-labeled BMSCs were not completely absent in the anti-CXCR4 group. Thus, other cytokines may also be important regulators of BMSC migratory behavior. Several studies have revealed that the macrophage chemoattractant protein-1 (MCP-1) (33) and hepatocyte growth factor (HGF) (34) are able to promote MSC migration into malignant gliomas, confirming the potential applications of genetically modified MSCs for cancer gene therapy. Xiao *et al* (35) also reported that TNF-α could enhance the ability of BMSCs to migrate into ischemic tissues.

Our results demonstrated that transplanted BMSCs reduce edema, inflammation and serum amylase activity in the injured pancreas and act to improve necrosis. There are numerous inflammatory factors involved in the progression of SAP, which trigger the activation of inflammatory cascades and may ultimately result in fatality. A recent study revealed that following BMSC transplant into SAP animal models, pro-inflammatory cytokine levels significantly reduced, while anti-inflammatory cytokines levels increased. They determined that this effect facilitated the control of pancreatic inflammation and repair of the damaged tissues (7). Furthermore, Yang *et al* (8) postulated that the paracrine factors of MSCs are important in cellular differentiation and thus in the repair processes of SAP. Our findings revealed the expression of numerous tubular complexes in the damaged pancreas when BMSCs were transplanted. Several studies have indicated that tubular complexes represent the beginning of regeneration and repair in the injured pancreas (36). As a result, we have hypothesized that BMSCs may contribute to pancreatic repair by stimulating the generation of tubular complexes, however the exact mechanism underlying this effect remains elusive and requires further investigation.

In summary, our findings confirmed that damaged pancreatic tissues in AP exhibit enhanced SDF-1 expression, and that the SDF-1/CXCR4 axis promotes the migration of transplanted BMSCs to facilitate in the reparative process that combat the disease.
